# Digging for Literature on Tailoring Cultural Offers With and for Older People From Ethnic Minority Groups: A Scoping Review

**DOI:** 10.1002/lim2.70004

**Published:** 2024-11-29

**Authors:** Stephanie Tierney, Jordan Gorenberg, Marta Santillo, Debra Westlake, Geoffrey Wong, Kerryn Husk, Sofia Vougioukalou, Ruthanne Baxter, Shoba Dawson, Nia Roberts, Caroline Potter, Harriet Warburton, Beth McDougall, Johannah Latchem, Kamal R Mahtani

**Affiliations:** ^1^ Nuffield Department of Primary Care Health Sciences University of Oxford Oxford UK; ^2^ Peninsula Medical School University of Plymouth Plymouth UK; ^3^ School of Medicine Cardiff University Cardiff UK; ^4^ University of Edinburgh Library University of Edinburgh Edinburgh UK; ^5^ School of Medicine and Population Health University of Sheffield Sheffield UK; ^6^ Libraries and Museums University of Oxford

**Keywords:** cultural provision, ethnic minority groups, older people, scoping review

## Abstract

**Introduction:**

Social prescribing addresses non‐medical issues (e.g., loneliness, financial worries, housing problems) affecting physical and/or mental health. It involves connecting people to external support or services, including ‘cultural offers’–events, groups and activities run within or by cultural organisations. Such offers need to be acceptable and accessible to diverse populations if forming part of a social prescription.

**Methods:**

A scoping review was conducted to identify what existing literature, conducted in the United Kingdom, tells us about tailoring cultural offers for older people (aged 60+ years) from ethnic minority groups. Relevant literature was searched for on electronic databases, through Google, via a questionnaire to cultural organisations and by contacting the study's advisory group.

**Results:**

Screening of 906 references–59 of which were read as full documents–resulted in six sources being included in the review. Some cultural activities described within them were run in traditional cultural spaces (e.g., museums, art galleries). Others were held in community centres. Data suggested that attending with others could reduce concerns about belonging. Barriers to engagement included low energy, language, poor confidence, accessing transport and unfamiliarity with a setting and/or activities. Provision of familiar food could help make people feel welcomed.

**Conclusions:**

Reviewed papers showed that consulting with target groups is important to ensure that activities are inclusive and sympathetically delivered. The review also highlighted a paucity of published research on the topic; this means that cultural providers have little evidence to draw on when developing cultural offers for older people from ethnic minority groups.

## 1 Introduction

People often go to see their general practitioner (GP) with problems that cannot be addressed with tablets or medical intervention [1]. For example, loneliness can lower people's mood, worries about finances may cause stress or work‐related anxiety can affect sleep. Social prescribing recognises that such ‘non‐medical’ issues impact health and well‐being, compromising health behaviours (e.g., diet and physical activity). It aims to assist people by connecting them to support or services, often in voluntary or community settings [2, 3]. This connecting work tends to be undertaken through meeting with someone called a link worker (other terms such as social prescriber or community connector may be used [4]).

As part of social prescribing, people may be connected to ‘cultural offers’–activities/groups with a cultural focus, provided in a cultural setting or at an organisation in the community. The range of activities that could be classed as thus highlights the diverse manner in which the term ‘cultural’ is understood [5]; a report by the All‐Party Parliamentary Group on Arts, Health and Wellbeing [6] described it as human creativity covering visual and performing arts, crafts, dance, film, literature, music and singing, and gardening. Evidence stresses the positive benefits that can transpire from arts and cultural engagement [6, 7, 8]. Hence, the cultural sector represents a potentially key resource for link workers to refer people to as part of a social prescription [9, 10].

Evidence around social prescribing involving people from ethnic minority groups is limited, highlighting a need for more access to such support for diverse communities [11]. People from ethnic minority groups may perceive as culturally inappropriate activities that form part of social prescriptions [12]. This idea around appropriateness of interventions has been debated within healthcare to explain why individuals may be disposed (or not) to use certain services [13, 14, 15]. Appropriateness can encompass the following:

· *Availability*: for example, the existence of cultural provision developed with or for people from ethnic minority groups and how information about such provision is publicised.
· *Accessibility*: for example, challenges that occur in how someone connects with or arrives at cultural provision (in‐person or online); such challenges may have to do with physically getting somewhere or gaining entry to a resource (including transport, technology or administrative/bureaucratic hurdles).
· *Acceptability*: for example, cultural provision offered so it fits with people's needs, values and preferences, that is, delivered in a respectful and culturally sensitive manner.
· *Affordability*: for example, addressing financial limitations that deter people from using cultural provision and those experienced by cultural providers and their capacity to offer a range of activities.

Social prescribing can be particularly relevant for people as they age, when they may (a) experience loneliness, (b) live with multiple health conditions affecting their mobility or mood, (c) have a low income and face economic challenges and (d) encounter a change in their levels of confidence and sense of purpose once they no longer work [16, 17, 18, 19, 20]. Understanding how to develop appropriate cultural offers for older people from ethnic minority groups is crucial if such provision is to form part of a social prescription for this population. Hence, we are conducting a study called TOUS–Tailoring cultural Offers with and for diverse older Users of Social prescribing. It involves the following elements:

· Identifying what is already known about cultural offers in the United Kingdom for or used by older people from ethnic minority groups, through reviewing existing literature on this topic (the focus of this paper).
· Mapping what is provided by the cultural sector in the United Kingdom for or accessed by older people from ethnic minority groups (which could form part of a social prescription). This has involved inviting cultural providers/groups/organisations to complete a short questionnaire.
· Exploring what we can learn from cultural organisations that have tried to provide cultural offers for and/or with older people from ethnic minority groups. This will involve spending time at organisations that completed the questionnaire referred to above.

## 2 Materials and Methods

### 2.1 Aims of the Review

A previous review we conducted in 2020–2021 sought to understand how, for whom, in what ways and why the cultural sector, as part of social prescribing, can improve the well‐being of older people (aged 60 years and above) [21]. Building on this previous research, a scoping review has been completed (and is described below) to explore the question: *What does the existing literature tell us about designing and running social prescribing cultural offers that are appropriate for older people (aged 60+ years) from ethnic minority groups in the* United Kingdom*?* It was conducted in accordance with the Joanna Briggs Institute (JBI) methodology for scoping reviews [22]. Through the review, we sought to:

· Capture what approaches/strategies have been used to create cultural provision that could form part of a social prescription for older people from ethnic minority groups, with a focus on what is known in relation to availability, accessibility, acceptability and affordability.
· Identify if these approaches/strategies have been evaluated and, if so, what outcomes have been reported.
· Ascertain which ethnic minority groups have been targeted.
· Ascertain if these approaches have been part of social prescribing/used by link workers.
· Highlight areas for further research.

### 2.2 Inclusion/Exclusion Criteria for the Review

· *Participants*: Older people from ethnic minority groups [23, 24]–who would constitute an under‐served population in research [25]. To be included, the average age of participants in a study/report had to be 60+ years (based on how the World Health Organization has defined older adults). We initially stated that publications had to include at least 50% of older people from ethnic minority groups. However, as the review progressed, in order to avoid an ‘empty’ review, with no included publications, we broadened the inclusion criteria to involve at least 15% of participants from an ethnic minority group.
· *Concept*: Cultural provision that could (or does) form part of a social prescription. Based on definitions from key organisations (e.g., the All‐Party Parliamentary Group on Arts, Health and Wellbeing, the United Nations Educational, Scientific and Cultural Organization, the European Commission, the Department for Digital, Culture, Media and Sport), we considered cultural provision in the following broad areas:
  ○ Heritage (e.g., museums, historical venues, historical buildings, curated green spaces),
  ○ Performance (e.g., music, theatre, dance),
  ○ Visual arts (e.g., photography, crafts, fine art),
  ○ Books/literature (e.g., poetry, libraries, creative writing),
  ○ Audio‐visual (e.g., films, podcasts, radio),
  ○ We did not include sports or faith groups/churches; these are broad topics that require dedicated research. We did not include recreational activities (e.g., gambling, theme parks, video games) as link workers are unlikely to refer to such support as part of social prescribing. Reading/book groups were included but bibliotherapy was excluded as this is a more therapeutic approach.
· *Context*: The review focused on cultural provision delivered in the United Kingdom and publications written in English.
· *Types of sources*: This review considered experimental and quasi‐experimental study designs including randomised controlled trials, non‐randomised controlled trials, before and after studies and interrupted time series studies. In addition, analytical observational studies including prospective and retrospective cohort studies, case‐control studies and cross‐sectional studies were considered, as were qualitative and mixed methods studies. Systematic reviews were excluded but their reference lists were checked for additional primary studies. We did not include conference abstracts, although we contacted their authors to see if they had published a full set of findings. We did not include discussion or commentary papers or theses/dissertations. We did a search for reports/evaluations–details about searching for grey literature are provided below.

### 2.3 Search Strategy

The following databases were searched in September 2023 (no time limit for when papers were published was used):

· Medline (OvidSP)
· CINAHL (EBSCOHost)
· Social Sciences Citation Index and Arts and Humanities Citation Index (Web of Science Core Collection)
· Applied Social Sciences Index and Abstracts (ProQuest)
· PsycINFO (OvidSP)

Search terms were tailored for each database with support from an information specialist (NR) (see Supporting Information File S1 for an example search on Medline). Terms for older people and studies from the United Kingdom were based on previously published work [26, 27]. The reference lists of all included sources were screened for additional, relevant studies.

To identify grey literature (research or evaluations falling outside traditional academic journals such as reports from organisations/governments), we undertook a search on Google in November 2023. Three searches were conducted on Google using different site or domain limiters–gov.uk; ac.uk; org.uk. The following search terms were used: (“ethnic minority” OR “ethnic minorities” OR BAME) AND (“older people” OR elderly OR “older adults”) AND (heritage OR museum* OR dancing OR singing OR music OR libraries OR theatre* OR art OR arts). We looked at the first 100 references returned from each of these searches (300 in total).

Alongside the Google searches, for the mapping exercise for the TOUS study mentioned above, we invited organisations or groups responding to our questionnaire to forward reports/evaluations; we know this can be an important means for identifying research or reports related to social prescribing [3, 28]. We also invited members of the study's advisory group (composed of people delivering social prescribing and cultural sector representatives) to provide us with potentially relevant references.

### 2.4 Screening

Following the deduplication of search results in Endnote and the removal of studies with non‐UK nations in the title, all identified citations were entered into Rayyan, an online platform that assists with managing references in a systematic review. Titles and abstracts of these citations were screened on Rayyan by one researcher (JG) against the inclusion/exclusion criteria for the review. A second reviewer (ST or MS) independently screened 20% of these references to check for systematic errors. Potentially relevant papers, or when there was insufficient information to make this decision, were retrieved in full and read. Full texts of selected citations were assessed in detail against the inclusion criteria by two researchers (JG or MS). Any disagreements between reviewers were resolved through discussion, or with, input from an additional reviewer (ST).

### 2.5 Data Extraction

Data were extracted from included papers by one reviewer (JG or MS) into an Excel table developed by the review team. Extracted data included specific details about the participants, type of cultural provision and how it was developed, where it was delivered (part of the country), study methods (data collection and analysis). As critical appraisal is not a common component of scoping reviews, it was not undertaken.

### 2.6 Data Synthesis and Presentation

Extracted data were used to summarise, narratively, data extracted from included papers and to produce tables presented below.

## 3 Results

**FIGURE 1:**
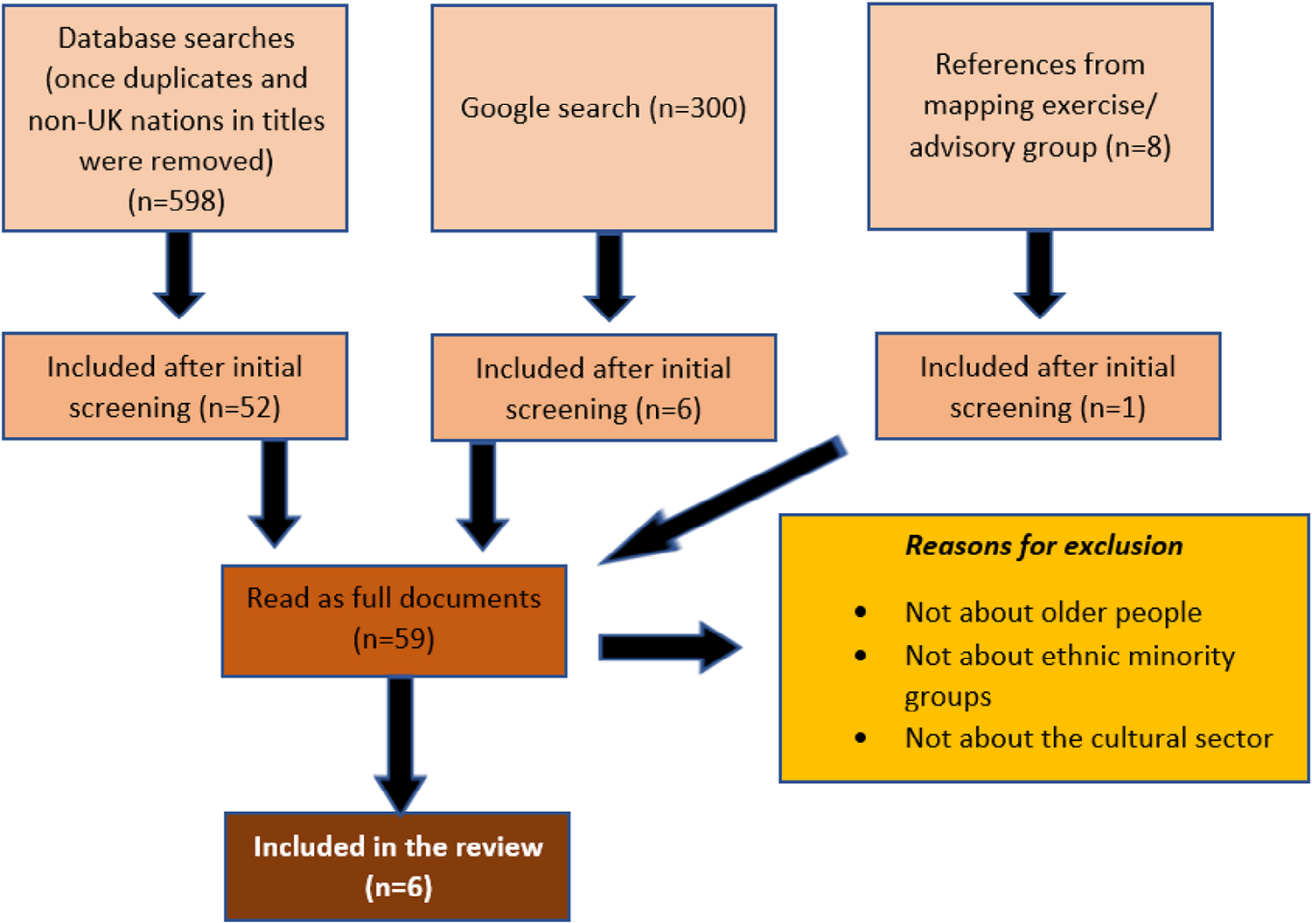
Flowchart of searching and screening.

As indicated in Figure 1, the scoping review involved screening a total of 906 items, sourced from databases (*n* = 598), a Google search for grey literature (*n* = 300) and references obtained from the mapping exercise questionnaire/advisory group (*n* = 8). Following the screening of titles and abstracts, 59 references were read as full‐texts and six were included in the scoping review. The majority of references were excluded because they did not include older people from ethnic minority groups or did not focus on the cultural sector or activities.

### 3.1 Overview of the Included Sources

As outlined in Table 1, three academic papers and three reports were included. They involved older people who ranged in age from 60 to 94 years. In terms of ethnic minority groups, South Asian, Chinese, African and Caribbean communities were represented, although details of specific populations involved were not always provided (see Table 1). Study designs varied, with two papers employing qualitative methods and one using a mixed‐methods approach (see Table 1). Included reports contained limited details on data collection or analysis. Three documents described working with specific populations (aside from ethnic minority groups): individuals with dementia [33], culturally inactive older people [29] and older adults at risk of loneliness and social isolation [34].

**TABLE 1:** Characteristics of included sources.

Author and source	Aim of study/report	Part of the UK covered	Research design	Sample	Data collection	Analysis
Goulding [29] Journal article	To understand how visiting contemporary art galleries and discussing the artwork affected culturally inactive participants’ social and cultural capital	North East England	Qualitative	Nineteen participants aged 64 and older; 16% were defined as non‐White	Interviews and focus groups	Constant comparison
Goulding [30] Journal article	To explore what social contexts bring to the experience of participation and how ageing produces material, physical and relational constraints to cultural participation	North East England	Qualitative	Forty participants aged 60–91 years; 22.5% non‐White: six were South Asian and three were Chinese	Interviews	Constant comparison
Hackney Caribbean Elderly Organisation [31] Report	To report on an organisation that seeks to enhance the quality of life and well‐being of older people from an African and Caribbean background by preventing isolation and building connections	London	Unclear	One hundred twenty Caribbean older people	Unclear	Unclear
Lowe [32] Report	To report on projects that set out to engage older populations seen as underserved–the first four focused on attracting older people from an ethnic minority group	London and Manchester	Unclear	African Caribbean, Chinese, South Asian–one case study referred to ethnic minority groups	Unclear	Unclear
Lynch [33] Report	To explore if multi‐art form artistic practice can help reduce stigma associated with dementia in South Asian communities and how culturally specific it needs to be	Havering, Hounslow, Leeds, Leicester, Slough and Tower Hamlets	Unclear (although seemed to be qualitative)	One hundred fourteen women aged 40–80+ and nine men 70+, including second‐generation women born in the United Kingdom, people from India, Bangladesh, Pakistan and Sri Lanka	Interviews	Unclear
Thomson et al. [34]. Journal article	To assess changes in psychological well‐being for older adults engaged in museums on prescription	London and Kent	Mixed methods	One hundred fifteen participants aged 65–94 years; 18% defined as non‐White	Questionnaires and interviews, alongside weekly reflective diaries	Multivariate analysis of variance; qualitative data not presented as themes

### 3.2 Information About Cultural Provision for Older People From Ethnic Minority Groups

As shown in Table 2, the reviewed studies and reports offer insights into the development and evaluation of cultural provision for older people from ethnic minority backgrounds in the United Kingdom. These endeavours encompassed a range of activities, including visits to art galleries and museums, participatory theatre experiences, arts and crafts sessions and community‐based events.

**TABLE 2:** Cultural provision covered in sources included in the review.

Author	Type of cultural provision
Goulding [29]	Facilitated group sessions for older people to discuss contemporary artwork, with transport provided
Goulding [30]	Older people with different levels of engagement in creative/cultural activities–including visiting art galleries/museums, photography, painting, knitting, music concerts, reading, choirs, film group
Hackney Caribbean Elderly Organisation [31]	Mixture of activities: movement to the music session, arts and crafts, innovative arts and involvement in cultural events (e.g., some members worked with young people to contribute to a Carnival showcase at the local town hall)
Lowe [32]	The four case studies focused on ethnic minority groups involved: (a) music, visual art, food and object handling; (b) food, script writing and printing; (c) dance; (d) performance/theatre
Lynch [33]	Participatory theatre–involving multi‐sensory and non‐verbal ways to engage with people who have dementia
Thomson et al. [34]	Seven museums provide sessions as part of a social prescribing programme for older people (a 10‐week programme for 2 h a week)–sessions included curator talks, behind‐the‐scenes tours, object handling and discussion, and art activities inspired by the exhibits

Cultural provision described in included sources aimed to address social isolation and enhance well‐being. Of the academic papers, two were qualitative, focusing on theme development (related to social and cultural capital). The other assessed emotional well‐being using the Museum Wellbeing Measure for Older Adults [10]. The three reports did not use specific assessment tools to evaluate this provision. Cost‐effectiveness was not examined in any included document.

Extracted data (see Table 3) showed that engagement could be hindered by various barriers such as language constraints and cultural differences, emphasising a need for inclusive approaches. We looked for information about the involvement of older people from ethnic minority groups in developing the cultural provision/activities; three of the included sources made a reference to this. Lynch [33] described the creation of theatre with older people and engaging with individuals from the Indian, Pakistani, Bangladeshi and Sri Lankan diaspora. Likewise, Lowe [32] highlighted activities in which older people were consulted about what was important in terms of delivery of cultural provision. The report from Hackney Caribbean Elderly Organisation [31] suggested that provision there was tailored to the needs and requirements of the community and was co‐produced. However, there was a lack of detail in any document around specific strategies to make cultural provision attractive and acceptable to older people from ethnic minority groups. Only two of the included sources [31, 34] mentioned explicit involvement of social prescribers/link workers in referrals.

**TABLE 3:** Information about steps taken to provide inclusive, culturally sensitive offers in included sources.

Authors	What the source tells us about accessibility, acceptability, availability and/or affordability of cultural provision for older people from ethnic minority groups	Space where cultural provision was held
Goulding [29]	Highlighted that simply viewing an art exhibit is unlikely to enhance well‐being. Discussion about the art was a crucial component of cultural engagement, reducing psychosocial barriers to inclusion. Art was an avenue for reminiscing with others in the group. Having a facilitated experience encouraged people to ask questions and fostered self‐confidence. Being in a group was a means to social capital development; participants who attended on their own or with a family member did not develop the same connections/bonds with others.	Three contemporary art galleries were involved. Some participants felt strength in numbers from being in a group; they would not have felt confident being there alone (e.g., due to education level/socioeconomic status).
Goulding [30]	Suggested that cultural capital gained through life can shape cultural engagement. Lack of knowledge of cultural venues and access to them could be supported through facilitated visits. However, energy might be required to walk around settings, and access may be compromised if caring for a partner. A lack of confidence stopped women from ethnic minority groups from going out alone, especially when English was a second language. Linguistic barriers were also alluded to in terms of enjoying listening to music in one's own language.	Various–public spaces and home‐based.
Hackney Caribbean Elderly Organisation [31]	The organisation described in the report offered a range of activities–some were culturally specific such as Quadrille dancing, Windrush celebrations, and visiting the Victoria and Albert Museum for an African fashion exhibition. Food provided was familiar and acceptable to service users: “Caribbean foods–yam, banana, dumplings, chicken” (p. 8).	A community centre that is wheelchair accessible, which can provide food and hold a range of activities. It was described as an inclusive space, sensitive and person‐centred, where individuals could share life.
Lowe [32]	Noted that older people from ethnic minority groups should not be seen as homogeneous. The case studies showed how communities were consulted to find ways to make provision inclusive. Also, the idea was proposed of touring provision developed in one area for similar communities in other parts of the country.	The four case sites were in (a) a public research centre specialising in the history of London, (b) a community centre dedicated to the well‐being of ethnic minority communities, (c) four community centres and (d) care homes, day centres and community groups.
Lynch [33]	Emphasised that engaging with services for older people with dementia from a South Asian background could be hampered by communication/language barriers, transport, trust and stigma. The report mentioned that content for cultural activities needed to acknowledge cultural diversity relating to art forms and genres. It noted how cultural organisations may struggle to engage with care homes and to speak to a manager, who could take some convincing about the relevance of participatory theatre to residents (gatekeeping by care home managers as a specific barrier). The report referred to the importance of faith and food when trying to meet the needs of older people with dementia. Music associated with faith and festivals was also mentioned; songs used at places like dementia cafes and day centres were not necessarily those that people from a South Asian background would grow up knowing. Also highlighted the dangers of reminiscence work with people who experienced trauma from events such as Partition and racism in the United Kingdom.	Attempted to work with care homes that catered for older people from a South Asian background. It was difficult to gain access and trying to do so could be time‐consuming. Might involve several telephone calls and visiting in advance to build trust and relationships.
Thomson et al. [34]	Quantitative findings showed an improvement in all six areas that were measured (absorbed, active, cheerful, enlightened, encouraged and inspired) pre–post at three time points–start, mid and end of the 10‐week programme. These results were not specific to ethnic minority groups. Qualitative data suggested that participants enjoyed learning new information and skills. They liked being intellectually challenged through engaging with museum objects and having their thoughts listened to. Some participants suggested they would visit museums more often now, after the facilitated visits, but this was not specific to people from ethnic minority groups.	Museum settings allowed people to engage with objects and artefacts. They felt privileged to be able to do so but could find it tiring having to walk around these spaces.

## 4 Discussion

Documents included in the review highlighted that engaging in cultural offers in a group enables older people from ethnic minority groups to forge connections, feel supported and can foster their self‐confidence. However, being with and interacting with others may require additional energy which along with physical limitations experienced with ageing, needs to be considered. Other potential barriers to older people from ethnic minority groups engaging in cultural activities, identified in reviewed sources, include language needs, transport issues and the type of food provided.

A key finding was a paucity of published or accessible literature that focused on older ethnic minority populations and cultural provision in the United Kingdom. We located some documents that centred on older people but these lacked information on the inclusion of individuals from an ethnic minority group. Others were rejected because they centred on ethnic minority groups but lacked information on age or spanned generations. This paucity of literature means a lack of evidence for providers in the United Kingdom to draw on to optimise development and delivery of cultural provision for older people from ethnic minority groups. A failure to identify more sources may reflect:

· A lack of capacity to write up what is taking place, especially among smaller cultural providers. When funding is precarious, energy may be spent on developing grant applications instead. Evidence of impact in the form of an evaluation or report may be needed to secure further funding but these reports may not be published/openly available. It should be noted that a lack of documentation does not mean a lack of practice; we know from our mapping exercise for the wider TOUS study (see above) that cultural providers are creating offers with or for older people from ethnic minority groups.
· A lack of separation, among ethnic minority groups, around cultural provision in terms of age. For example, stakeholders have told us that cultural activities around Carnival or Mela are likely to be as part of a mixed‐age group.
· Talking to stakeholders associated with this project, among ethnic minority groups there may be less demarcation of cultural engagement from other activities and linking this to specific venues (e.g., going to a museum or library); rather, cultural provision forms part of broader activities bringing together food, music and dance.
· Definitions of ‘culture’ are socially constructed and have traditionally been formed by majority white cultural reference points. Cultural venues and organisations can be seen as spaces that bring people together to engage in activities that engender well‐being. However, they may be seen as inaccessible–both physically and in terms of somewhere that people feel comfortable. A feeling of belonging may be hampered when people are unable to identify with the activities offered. It should also be noted that acceptability of cultural offers may be tainted by centuries of colonialism and a feeling that settings (e.g., museums, libraries) are not a sphere for people from non‐white backgrounds [35, 36, 37, 38, 39, 40]. Hence, where activities are offered may shape how far they are perceived to be acceptable.

There was limited evidence in reviewed papers of active co‐production with ethnic minority groups, which can contribute to a disconnect between cultural offers and the preferences, needs and experiences of older ethnic minority individuals. Consequently, there is a risk that these initiatives do not effectively address the unique social, cultural and linguistic factors influencing participation and engagement by these communities [41].

### 4.1 Implications for Practice

Tailoring cultural offers to be inclusive and accessible is important so that people from a range of backgrounds can benefit from engaging. However, the ability of organisations or groups to tailor, when funding is insecure, may be limited. Reviewed sources proposed that by actively collaborating with older individuals from diverse ethnic backgrounds, institutions can develop offers that are relevant and acceptable. This is important for social prescribing; link workers need a range of provisions to be able to connect people as the same cultural offer will not be suitable for everyone. It is essential for link workers to be aware of the specific cultural needs and preferences of older people from ethnic minority groups and to facilitate access to culturally appropriate programmes and activities. Collaboration between cultural institutions, link workers and community organisations is key in this respect [42] to ensure that older ethnic minority individuals have equitable access to culturally enriching experiences that promote their well‐being.

Sources included in the review did identify barriers for providers to consider when trying to attract older people from ethnic minority groups to cultural offers. These include physical exhaustion (from walking around a venue), language, poor confidence, accessing transport and unfamiliarity with a setting and/or activities. Facilitated visits, with transport arranged, and the provision of culturally acceptable food, may help individuals to feel welcomed and safe in an unfamiliar space.

### 4.2 Implications for Future Research

Our scoping review highlighted a significant gap in the literature regarding cultural provision tailored for and/or with older people from ethnic minority groups. Moving forward, researchers should prioritise exploring the preferences of diverse populations to ensure inclusivity and effectiveness in cultural programming. Additionally, there is a need for more rigorous evaluations of existing cultural initiatives targeting older ethnic minority groups to determine their effectiveness and how they have an impact. By addressing this research gap, future studies can contribute valuable insights to inform the creation and implementation of culturally sensitive and inclusive programmes.

Of those documents that were located and included in the review, details on data collection and use of economic evaluations were lacking. Hence, more research is required covering affordability of tailoring to both organisations and older people.

### 4.3 Strengths and Limitations

The review only included studies and reports on ethnic minority groups in the United Kingdom. Adopting a broader search, country‐wise, may have identified further references. However, this review was produced to inform primary data collection as part of the TOUS study, which is taking place in the United Kingdom; the needs of ethnic minority groups in other countries may differ. A lack of studies from the United Kingdom reflects a difficulty that link workers may have in locating appropriate cultural support for older people from ethnic minority groups, even though the United Kingdom is at the forefront of social prescribing delivery. We acknowledge that ‘ethnic minority groups’ is a broad category, and that challenges for specific communities may vary accordingly. This is something that future research can explore.

## 5 Conclusion

This scoping review emphasises a limited evidence base in the United Kingdom to inform the provision of cultural offers that are accessible and acceptable for older people from ethnic minority groups. More research in this area is required, especially if cultural provision is to form part of a social prescription for this population. By uncovering this evidence gap, the review is a valuable starting point for future research endeavours aimed at enhancing cultural provision and social prescribing practices for older ethnic minority populations. While some of the cultural provision described in included documents holds promise in promoting social inclusion and well‐being among older ethnic minority populations, further research and evaluation are imperative to ensure their availability, accessibility, acceptability and affordability within the context of social prescribing initiatives.

## Conflicts of Interest

The authors declare no conflicts of interest.

## Ethics Statement

The authors have nothing to report.

## Consent

As this was a review, primary data were not collected from patients so no consent statement was required.

## Permission to Reproduce Material From Other Sources

The authors have nothing to report.

## Clinical Trial Registration

The authors have nothing to report.

## Supporting information



Supporting Information

## Data Availability

As this is a systematic review, papers/reports referred to, from which data were extracted, can be accessed by others.
