# Supplementary material for: Digging for Literature on Tailoring Cultural Offers With and for Older People From Ethnic Minority Groups: A Scoping Review
Source: Lifestyle Med (Hoboken). 2024 Nov 29;6(1):e70004. doi: 10.1002/lim2.70004 (PMC11605778; doi:10.1002/lim2.70004)
Supplement: Supplementary file 1 — Supporting Information [file LIM2-6-e70004-s001.docx]

**Supplementary File 1: Example search on Medline**

| **Search number** | **Search terms used** | **Number of hits returned** |
| --- | --- | --- |
| 1 | social prescri*.ti,ab,kf. | 456 |
| 2 | (link? adj2 (worker? or practitioner? or staff or personnel or officer? or team?)).ti,ab,kf. | 352 |
| 3 | gardens/ or museums/ or public facilities/ or senior centers/ or exp "sports and recreational facilities"/ | 12767 |
| 4 | language arts/ or reading/ | 25999 |
| 5 | Libraries/ | 2534 |
| 6 | art/ or motion pictures/ or paintings/ or sculpture/ or literature/ or music/ or nature/ | 43021 |
| 7 | ((art? or cultur* or sport* or nature or park? or greenspace? or green space? or bluespace? or blue space?) adj5 prescri*).tw,kw. | 1844 |
| 8 | (arts or theatre? or theater? or live performance? or cinema? or museum? or galler*).tw,kw. | 42300 |
| 9 | (((heritage or historic*) adj3 (venue? or building? or house?)) or (national trust or english heritage) or (castle? or palace?)).tw,kw. | 2246 |
| 10 | (cultur* adj3 (offer* or event? or performance? or activit* or venue?)).tw,kw. | 18724 |
| 11 | (cultur* adj3 (sector? or group? or organi?ation? or charity or charities)).tw,kw. | 13018 |
| 12 | ((cultur* or art?) adj2 (engage* or participat*)).tw,kw. | 1558 |
| 13 | (recreation* adj3 (event? or activit* or venue? or park?)).tw,kw. | 5891 |
| 14 | (library or libraries).ti. or (librar* adj3 (communit* or local* or neighbo?rhood? or access* or proximit)).tw,kw. or ((literary or literature or book? or poetry) adj3 (group? or club? or program* or festival? or event?)).tw,kw. or (reading group? or reading club?).tw,kw. or ((literature or book? or poetry) adj3 reading).tw,kw. | 30069 |
| 15 | ((craft? or sewing or knit* or crochet* or tapestr* or embroider*) adj3 (group? or club? or class* or program*)).tw,kw. or craft?.ti. | 1780 |
| 16 | ((art or painting or sculpture or photography or photographic) adj3 (group? or club? or class* or program* or festival? or event?)).tw,kw. | 5160 |
| 17 | (((music* or singing or drama or dance or dancing) adj3 (group? or club? or class* or program* or festival? or event?)) or ((communit* or local* or neighbo?rhood?) adj2 choir*)).tw,kw. | 5130 |
| 18 | (park? or greenspace? or green space? or bluespace or blue space? or garden?).ti. or ((park? or greenspace? or green space? or bluespace or blue space? or garden?) adj5 (public or communit* or local* or neighbo?rhood? or national or urban? or access* or proximit*)).tw,kw. or ((countryside or nature) adj5 (access* or proximit*)).tw,kw. | 20844 |
| 19 | ((((community or day or senior*) adj2 (center? or centre?)) or community hall? or village hall?) and (art? or painting or sculpture or photography or photographic or craft? or sewing or knit* or crochet* or tapestr* or embroider* or music* or singing or drama or dance or dancing or garden?)).tw,kw. | 274 |
| 20 | or/1-19 | 218187 |
| 21 | exp aged/ | 3462931 |
| 22 | elderly.tw,kw. | 294508 |
| 23 | geriatric*.tw,kw. | 78162 |
| 24 | senior.tw,kw. | 42652 |
| 25 | (older adj (adult? or m#n or wom#n or person? or people)).tw,kw. | 182622 |
| 26 | exp geriatrics/ | 31553 |
| 27 | (Age? adj3 (over or older) adj2 (6# or 7# or 8# or 9#)).tw. | 43224 |
| 28 | (sexagenarian or septuagenarian or octogenarian or nonagenarian or centenarian).tw,kw. | 3525 |
| 29 | gerontolog*.tw,kw. | 8859 |
| 30 | (">=6# years old" or ">6# years old").tw. | 28014 |
| 31 | **(">=7# years old" or ">7# years old").tw.** | 16766 |
| 32 | (">=8# years old" or ">8# years old").tw. | 10123 |
| 33 | (">=9# years old" or ">9# years old").tw. | 3376 |
| 34 | 21 or 22 or 23 or 24 or 25 or 26 or 27 or 28 or 29 or 30 or 31 or 32 or 33 | 3666006 |
| 35 | ethnicity/ or racial groups/ | 93211 |
| 36 | exp african people/ or exp asian people/ or exp black people/ or caribbean people/ or exp european people/ or exp "middle eastern and north africans"/ | 179142 |
| 37 | Roma/ | 1062 |
| 38 | ethnic and racial minorities/ or minority groups/ | 18324 |
| 39 | culture/ or cross-cultural comparison/ or cultural characteristics/ or exp cultural diversity/ | 86964 |
| 40 | multilingualism/ | 5977 |
| 41 | communication barriers/ or limited english proficiency/ | 7415 |
| 42 | exp "emigrants and immigrants"/ or refugees/ or "transients and migrants"/ | 40779 |
| 43 | (((ethnic or racial or minority) adj2 (group? or population? or people? or adult? or senior?)) or ethnicity).tw,kw. or (ethnic* or race or racial or minorit*).ti. | 180681 |
| 44 | (afrocaribbean? or afro-caribbean? or black british or british african? or british caribbean? or (black adj2 (population? or people or adult? or communit*))).tw,kw. | 10530 |
| 45 | (asian? or indian? or pakistani? or bangladeshi? or sri lankan? or afghan* or nepalese or nepali or chinese).tw,kw. | 549388 |
| 46 | ((mixed adj (race or heritage)) or biracial).tw,kw. | 1999 |
| 47 | (roma? or (traveller adj2 (population? or people or adult? or communit*))).tw,kw. | 6467 |
| 48 | (immigrant? or migrant? or refugee?).tw,kw. | 63266 |
| 49 | diversity.ti. or (diversity adj5 (inclusion or exclusion or equality or inequality or equity or inequity)).tw,kw. or (divers* adj3 (population? or people or adult? or neighbo?rhood? or communit*)).tw,kw. | 114494 |
| 50 | (mother tongue or ((first or 1st or second or 2nd or dominant or preferred) adj2 language?) or ((language or english) adj2 (proficien* or fluen* or barrier?)) or (bilingual* or bi-lingual* or multilingual* or multi-lingual*) or sign language or ((welsh or gaelic or cornish) adj5 (speak* or language))).tw,kw. | 23456 |
| 51 | ((cultural* adj3 (adapt* or tailor* or barrier?)) or (crosscultur* or cross-cultur*)).tw,kw. | 28471 |
| 52 | or/35-51 | 1096014 |
| 53 | 20 and 34 and 52 | 1755 |
| 54 | exp United Kingdom/ | 391095 |
| 55 | (national health service* or nhs*).ti,ab,in. | 274738 |
| 56 | (english not ((published or publication* or translat* or written or language* or speak* or literature or citation*) adj5 english)).ti,ab. | 50507 |
| 57 | (gb or "g.b." or britain* or (british* not "british columbia") or uk or "u.k." or united kingdom* or (england* not "new england") or northern ireland* or northern irish* or scotland* or scottish* or ((wales or "south wales") not "new south wales") or welsh*).ti,ab,jw,in. | 2469987 |
| 58 | (bangor or "bangor's" or cardiff or "cardiff's" or newport or "newport's " or st asaph or "st asaph's" or st davids or swansea or "swansea's").ti,ab,in. | 71141 |
| 59 | (aberdeen or "aberdeen's" or dundee or "dundee's" or edinburgh or "edinburgh's" or glasgow or "glasgow's" or inverness or (perth not australia*) or ("perth's" not australia*) or stirling or "stirling's").ti,ab,in. | 259955 |
| 60 | (armagh or "armagh's" or belfast or "belfast's" or lisburn or "lisburn's" or londonderry or "londonderry's" or derry or "derry's" or newry or "newry's").ti,ab,in. | 34276 |
| 61 | (bath or "bath's" or ((Birmingham not alabama*) or ("birmingham's" not alabama*) or bradford or "bradford's" or brighton or "brighton's" or bristol or "bristol's" or carlisle* or "carlisle's" or (cambridge not (massachusetts* or boston* or harvard*)) or ("cambridge's" not (massachusetts* or boston* or harvard*)) or (canterbury not zealand*) or ("canterbury's" not zealand*) or chelmsford or "chelmsford's" or chester or "chester's" or chichester or "chichester's" or coventry or "coventry's" or derby or "derby's" or (durham not (carolina* or nc)) or ("durham's" not (carolina* or nc)) or ely or "ely's" or exeter or "exeter's" or gloucester or "gloucester's" or hereford or "hereford's" or hull or "hull's" or lancaster or "lancaster's" or leeds* or leicester or "leicester's" or (lincoln not nebraska*) or ("lincoln's" not nebraska*) or (liverpool not (new south wales* or nsw)) or ("liverpool's" not (new south wales* or nsw)) or ((london not (ontario* or ont or toronto*)) or ("london's" not (ontario* or ont or toronto*)) or manchester or "manchester's" or (newcastle not (new south wales* or nsw)) or ("newcastle's" not (new south wales* or nsw)) or norwich or "norwich's" or nottingham or "nottingham's" or oxford or "oxford's" or peterborough or "peterborough's" or plymouth or "plymouth's" or portsmouth or "portsmouth's" or preston or "preston's" or ripon or "ripon's" or salford or "salford's" or salisbury or "salisbury's" or sheffield or "sheffield's" or southampton or "southampton's" or st albans or stoke or "stoke's" or sunderland or "sunderland's" or truro or "truro's" or wakefield or "wakefield's" or wells or westminster or "westminster's" or winchester or "winchester's" or wolverhampton or "wolverhampton's" or (worcester not (massachusetts* or boston* or harvard*)) or ("worcester's" not (massachuse tts* or boston* or harvard*)) or (york not ("new york*" or ny or ontario* or ont or toronto*)) or ("york's" not ("new york*" or ny or ontario* or ont or toronto*))))).ti,ab,in. | 1765108 |
| 62 | 54 or 55 or 56 or 57 or 58 or 59 or 60 or 61 | 3099871 |
| 63 | (exp africa/ or exp americas/ or exp antarctic regions/ or exp arctic regions/ or exp asia/ or exp oceania/) not (exp great britain/ or europe/) | 3347900 |
| 64 | 62 not 63 | 2933793 |
| 65 | 53 and 64 | 166 |
| 66 | limit 65 to english language | 165 |
